# Targeted Therapy for Severe Sjogren’s Syndrome: A Focus on Mesenchymal Stem Cells

**DOI:** 10.3390/ijms252413712

**Published:** 2024-12-22

**Authors:** Carl Randall Harrell, Ana Volarevic, Aleksandar Arsenijevic, Valentin Djonov, Vladislav Volarevic

**Affiliations:** 1 Regenerative Processing Plant, LLC, 34176 US Highway 19 N, Palm Harbor, FL 34684, USA; dr.harrell@regenerativeplant.org; 2 Department of Psychology, Center for Research on Harmful Effects of Biological and Chemical Hazards, Faculty of Medical Sciences, University of Kragujevac, 69 Svetozara Markovica Street, 34000 Kragujevac, Serbia; ana.volarevic@fmn.kg.ac.rs; 3 Departments of Genetics, Microbiology and Immunology, Center for Research on Harmful Effects of Biological and Chemical Hazards, Faculty of Medical Sciences, University of Kragujevac, 69 Svetozara Markovica Street, 34000 Kragujevac, Serbia; salvatoredjulijano@gmail.com; 4 Institute of Anatomy, University of Bern, Baltzerstrasse 2, 3012 Bern, Switzerland; valentin.djonov@unibe.ch; 5 Faculty of Pharmacy Novi Sad, Heroja Pinkija 4, 21000 Novi Sad, Serbia

**Keywords:** mesenchymal stem cells, primary Sjögren’s syndrome, autoimmune response, immunosuppression, targeted therapy

## Abstract

Primary Sjögren’s syndrome (pSS) is an autoimmune disease characterized by the infiltration of lymphocytes on salivary and lacrimal glands, resulting in their dysfunction. Patients suffering from severe pSS have an increased risk of developing multi-organ dysfunction syndrome due to the development of systemic inflammatory response, which results in immune cell-driven injury of the lungs, kidneys, liver, and brain. Therapeutic agents that are used for the treatment of severe pSS encounter various limitations and challenges that can impact their effectiveness. Accordingly, there is a need for targeted, personalized therapy that could address the underlying detrimental immune response while minimizing side effects. Results obtained in a large number of recently published studies have demonstrated the therapeutic efficacy of mesenchymal stem cells (MSCs) in the treatment of severe pSS. MSCs, in a juxtacrine and paracrine manner, suppressed the generation of inflammatory Th1 and Th17 lymphocytes, induced the expansion of immunosuppressive cells, impaired the cross-talk between auto-reactive T and B cells, and prevented the synthesis and secretion of auto-antibodies. Additionally, MSC-derived growth and trophic factors promoted survival and prevented apoptosis of injured cells in inflamed lacrimal and salivary glands, thereby enhancing their repair and regeneration. In this review article, we summarized current knowledge about the molecular mechanisms that are responsible for the beneficial effects of MSCs in the suppression of immune cell-driven injury of exocrine glands and vital organs, paving the way for a better understanding of their therapeutic potential in the targeted therapy of severe pSS.

## 1. Introduction

Primary Sjögren’s syndrome (pSS) is a chronic autoimmune disorder characterized by the infiltration of lymphocytes into exocrine glands, particularly the salivary and lacrimal glands, leading to their dysfunction [1]. Accordingly, dry eyes (xerophthalmia) and dry mouth (xerostomia) are the main clinical signs of pSS [2]. Patients usually experience a gritty sensation and redness of the eyes, although excessive tearing could be observed as a compensatory response [2]. Prolonged dryness can lead to corneal ulcers and meibomian gland dysfunction [2]. Difficulty swallowing, altered taste, and an increased risk of dental caries and fungal infections due to reduced saliva production are also frequently seen in patients with pSS [1,2]. Systemic manifestations of detrimental immune response include fatigue, arthritis, myalgia, lymphadenopathy, peripheral neuropathy, tubulointerstitial nephritis, and cognitive impairment [1,2].

The pSS is, according to clinical symptoms and the dissemination of inflammatory response, categorized into mild, moderate, and severe pSS [2,3]. Patients with mild pSS experience dry eyes and dry mouth without systemic inflammation [2,3]. They may manage symptoms with artificial tears and saliva substitutes and generally maintain a good quality of life. The increased severity of dryness, fatigue, arthralgia, and mild lymphadenopathy is usually observed in patients with moderate pSS [2,3]. Since symptoms interfere with daily activities, these patients might require medications for symptom relief, such as pilocarpine or cevimeline, which stimulate salivary gland function [2,3]. Severe pSS is characterized by severe xerophthalmia, chronic pain in the eyes, corneal ulcerations, xerostomia, salivary gland enlargement, multiple oral infections, severe fatigue, widespread arthritis, myalgia, vasculitis, and severe lymphadenopathy [3,4]. Patients suffering from severe pSS have an increased risk of developing life-threatening complications, particularly mucosa-associated lymphoid tissue (MALT) lymphoma and multiorgan dysfunction syndrome due to immune cell-driven damage of the lungs, kidneys, and liver [1,4]. Brain injury is extremely rare in patients with pSS [1]. Patients with severe pSS often experience debilitating symptoms, and their quality of life is markedly reduced [3,4].

Current therapeutic approaches for severe pSS include symptomatic treatment for dry eyes and dry mouth (such as the use of artificial tears, punctual plugs, anti-inflammatory eye drops, saliva substitutes, and medications such as pilocarpine or cevimeline) and immunomodulatory therapy for the attenuation of systemic immune cell-driven inflammation [5]. For this purpose, disease-modifying antirheumatic drugs (DMARDs), corticosteroids, immunosuppressive medicaments, and biologics are usually used [6]. They modulate the migration and activation of innate immune cells, inhibit the proliferation of auto-reactive T and B cells, down-regulate the production of autoantibodies, and prevent the deposition of immune complexes in small blood vessels, joints, and kidneys of patients with pSS, attenuating disease progression [5,6].

However, therapeutic agents that are used for the treatment of severe pSS encounter various limitations and challenges that can impact their effectiveness [5]. Artificial tears and saliva substitutes provide temporary relief but do not address the underlying autoimmune process [5,6]. Patients often need to use them multiple times a day, which can be inconvenient and insufficient for severe dryness [5,6]. While DMARDs are effective for the attenuation of musculoskeletal symptoms, they do not address dryness and usually have serious side effects after long-term use (liver toxicity, retinal damage, and bone marrow suppression) [6]. Corticosteroids and immunosuppressive drugs may effectively alleviate systemic inflammatory response, but their long-term use can lead to significant side effects, including osteoporosis, weight gain, diabetes, hypertension, and increased risk of infections due to prolonged immunosuppression [5,6]. These limitations highlight the need for more targeted, personalized therapies that could address the underlying mechanisms of severe pSS while minimizing side effects and improving overall patient quality of life [5,6].

Mesenchymal stem cells (MSCs) are self-renewable adult stem cells that possess potent regenerative and immunomodulatory characteristics [7]. They exhibit a fibroblast-like morphology and are defined by the expression of specific surface markers such as CD73, CD90, and CD105. MSCs do not express hematopoietic markers (CD34 and CD45) and human leukocyte antigen (HLA) class II proteins, making them an attractive option for allogeneic transplantation [7]. Furthermore, MSCs exhibit a plasticity that enables them to respond to various microenvironmental cues, guiding their differentiation pathways depending on the signals they encounter [7,8]. MSCs spontaneously differentiate into cells of mesodermal origin (adipocytes, chondrocytes, and osteocytes), but under appropriate culture conditions, MSCs may generate cells of ectodermal and endodermal origin [7,8]. Additionally, MSCs are immunoregulatory and angiomodulatory cells [7,8]. They produce various immunosuppressive proteins and microRNAs (miRNAs) capable of attenuating detrimental immune responses driven by innate immune cells’ auto-reactive T and B lymphocytes [7]. Moreover, MSC-derived growth factors and pro-angiogenic molecules could provide trophic support to injured cells of lacrimal and salivary glands, enhancing their repair and regeneration [8]. Results obtained in a large number of recently published experimental studies and pilot clinical trials have demonstrated the therapeutic efficacy of MSCs in the treatment of severe pSS [9,10]. Accordingly, in this review article, we summarized current knowledge about the molecular mechanisms that are responsible for the therapeutic potential of MSCs in the suppression of immune cell-driven destruction of salivary and lacrimal glands and for the attenuation of systemic inflammatory response, opening new avenues for the targeted therapy of severe pSS. An extensive literature review was carried out in August 2024 across three databases (MEDLINE, EMBASE, and Google Scholar) from 1990 to the present. Keywords used in the selection were “Primary Sjögren’s Syndrome”, “Sjogren’s disease”, “dry eyes”, “dry mouth”, “mesenchymal stem cells”, “targeted therapy”, “signaling pathways”, “inflammation”, “immune cells”, “immunosuppression”, “immunoregulation”, and “tissue repair and regeneration”. All journals were considered, and an initial search retrieved 378 articles. The abstracts of all these articles were subsequently reviewed by two of the authors (C.R.H. and V.V.) independently to confirm their relevance to the subject of this manuscript. Eligible studies had to delineate molecular and cellular mechanisms that are involved in the MSC-dependent attenuation of the autoimmune response in experimental models of pSS and in patients suffering from severe pSS, and their findings were analyzed in this review.

## 2. Molecular Mechanisms Responsible for the Development and Progression of pSS

The pathogenesis of pSS involves a multifactorial interplay of genetic, environmental, and immunological factors [11,12,13,14]. The presence of certain human leukocyte antigen (HLA) genes (HLA-DR3, HLA-DR4, HLA-DR52, HLA-DQ2, and HLA-DQ8) significantly increases the risk of developing pSS in genetically predisposed individuals [11]. These genes are responsible for the synthesis of HLA class II molecules that are expressed on professional antigen-presenting cells (dendritic cells (DCs), macrophages, and B lymphocytes), playing a crucially important role in the presentation of auto-antigens (Ro (SS-A) and La (SS-B) ribonucleoproteins) to T cells, initiating their activation and proliferation [11].

In addition to HLA genes, the presence of several “non-HLA” genetic variants creates a complex genetic background that influences susceptibility to pSS through mechanisms of immune deregulation [11]. Precisely, the presence of single nucleotide polymorphisms (SNPs) rs2004640 and rs10954213 in the Interferon Regulatory Factor 5 (IRF5) gene deregulates type I interferon response against Epstein–Barr virus (EBV) and cytomegalovirus (CMV). These components may mimic auto-antigens, initiating an auto-reactive immune response in patients with pSS [11,14]. The hallmark feature of pSS is the dense infiltration of T lymphocytes into the salivary and lacrimal glands [12]. The existence of SNPs rs7574865 in the Signal Transducer and Activator of Transcription 4 (STAT-4) gene and rs2476601 in Protein Tyrosine Phosphatase Non-Receptor Type 22 (PTPN22) gene affects the differentiation, activation, and cytokine production of CD4+ T helper (Th) and CD8+ cytotoxic T cells (CTLs) [11,12]. Th1 cell-derived IFN-γ enhances the expression of HLA molecules on professional antigen-presenting cells and facilitates the activation of other immune cells, perpetuating the inflammatory response [11,12]. Additionally, IFN-γ, secreted by Th1 cells, and IL-17, produced by Th17 cells, induce the activation of NOD-Like Receptor Family Pyrin Domain Containing 3 (NLRP3) gene and the nuclear factor kappa-light-chain-enhancer of activated B cells (NF-κB) in macrophages and neutrophils, inducing potent innate immune cell-driven inflammation [12,13]. The presence of rs35829419 in the NLRP3 gene and rs5029930 in the TNFAIP3 gene enhance NLRP3 and NF-κB-dependent signaling, aggravating macrophage and neutrophil-driven inflammatory response in salivary and lacrimal glands of patients with pSS [11,13]. The cross-talk between CD4+T cells and B cells is a critical aspect of the pSS’s pathogenesis, leading to the increased production of anti-Ro and anti-La autoantibodies and sustained inflammation [13,15]. Some activated CD4+T cells differentiate into follicular T helper cells (Tfh), which reside in germinal centers of lymphoid tissues and provide essential help to B cells through the expression of CD154 that binds to CD40 on B cells, facilitating their activation and isotype switching [13,15]. Additionally, Tfh produces IL-21 and IFN-γ, which promote B cell survival, proliferation, and differentiation into auto-antibody-secreting plasma cells [15]. The presence of SNPs rs703842 and rs951482 in the B cell Activating Factor (BAFF) gene and rs7528684 in the Fc Receptor-Like 3 (FCRL3) gene promotes the T cell-dependent activation of B cells and autoantibody production [11,15]. Importantly, autoreactive B cells can also influence T cell-driven inflammation in patients with pSS [15]. Activated B cells present auto-antigens to T cells and produce inflammatory cytokines (IL-6 and tumor necrosis factor-alpha (TNF-α)), which enhance T cell activation [13,15]. The persistent interactions between T and B cells can establish populations of memory T and B cells, which are able to respond rapidly upon re-exposure to auto-antigens, perpetuating the autoimmune process [16]. Additionally, the chronic inflammation and continuous interaction between auto-reactive T and B cells promote the formation of ectopic germinal centers in salivary and lacrimal glands [16]. These structures mimic secondary lymphoid organs and facilitate ongoing T:B cell crosstalk, resulting in the generation of a more robust autoimmune response [12,15,16].

CD8+CTLs also play an important pathogenic role in the development and progression of pSS [17]. As a result of molecular mimicry, CD8+CDLs react to viral antigens that share structural similarities with self-antigens in glandular tissues and directly attack epithelial cells in the glands, contributing to glandular atrophy and dysfunction [17]. Damage to epithelial cells in the salivary and lacrimal glands may enhance the expression of auto-antigens and may induce the increased synthesis of pro-inflammatory molecules (alarmins, damage-associated molecular patterns (DAMPs), and monocyte- and lymphocyte-attracting chemokines), creating a feedback inflammatory loop that exacerbates the autoimmune response [12,17].

In addition to the increased presence of inflammatory T and B cells, patients suffering from severe pSS usually have a reduced number of immunosuppressive cells (CD4+FoxP3+T regulatory cells (Tregs) and myeloid-derived suppressor cells (MDSCs)) which are essential for maintaining immune homeostasis and preventing excessive inflammation in injured lacrimal and salivary glands [18,19]. Tregs suppress T cell-driven inflammatory response by secreting immunosuppressive cytokines (IL-10 and transforming growth factor beta (TGF-β)) and by expressing inhibitory molecules (cytotoxic T-lymphocyte-associated protein 4 (CTLA-4) and programmed death receptor ligand (PDL)), which inhibit the production of inflammatory cytokines and induce apoptosis of autoreactive T cells [18]. Even when present in sufficient numbers, Tregs of patients with pSS usually exhibit functional impairments, such as a decreased production of IL-10 and TGF-β [18]. The existence of SNPs rs1800871 or rs1800872 in the interleukin IL-10 gene and rs1800470 or rs11568848 in the TGF-β1 gene down-regulates the production of these immunosuppressive cytokines, crucially contributing to the breakage of Treg-dependent immune tolerance in the lacrimal and salivary glands of patients with pSS [11,18]. A deficiency in Tregs function also leads to abnormal B cell activation, the increased production of anti-Ro and anti-La auto-antibodies, and results in the development of severe pSS [15,18].

In a similar manner to Tregs, MDSCs create an immunosuppressive environment in inflamed and injured lacrimal and salivary glands of patients with pSS and inhibit detrimental inflammatory and autoimmune responses [19]. MDSCs inhibit the expansion of T cells by altering the metabolic environment in glandular tissues through the depletion of essential nutrients (arginine and tryptophan) and by generating reactive oxygen species, which can inhibit T cell receptor signaling pathways [12,19]. Additionally, MDSCs down-regulate the expression of HLA molecules and co-stimulatory molecules on DCs and macrophages, reducing their ability to present antigens to T cells impairing T cell-driven adaptive immune responses [12,19]. MDSCs secrete high levels of immunosuppressive factors (IDO, IL-10, and TGF-β) that favor the differentiation of naïve T cells into Tregs, suppress the activation of autoreactive B cells, and attenuate the production of autoantibodies in plasma cells [15,19].

## 3. The Therapeutic Potential of MSCs in the Treatment of pSS

An important characteristic of MSCs that differentiates them from other adult stem cells is their ability to inhibit detrimental immune responses to suppress the expansion and activation of auto-reactive T and B cells, thereby alleviating the progression of autoimmune and inflammatory diseases [7]. MSCs can suppress the proliferation of activated immune cells in a juxtacrine manner (direct cell-to-cell contact) and by paracrine signaling, utilizing MSC-derived immunomodulatory factors [20]. The secretome produced by MSCs is rich in anti-inflammatory cytokines (such as IL-10, IL-35, TGF-β, and IL-1 receptor antagonist (IL-1Ra)), immunosuppressive proteins (indoleamine 2,3-dioxygenase (IDO), prostaglandin E2 (PGE2), soluble TNF receptor (sTNFR), heme oxygenase (HO)), and immunoregulatory miRNAs [21]. These molecules inhibit pro-inflammatory pathways by targeting crucial genes that regulate activation, differentiation, and cytokine production in inflammatory and auto-reactive immune cells [20,21]. Importantly, MSC-derived immunomodulatory factors can be delivered directly to target cells via MSC-derived exosomes (MSC-Exos), which are tiny extracellular vesicles that, due to their nano-size and lipid bilayer, can bypass all biological barriers in the body [22,23].

MSCs and MSC-Exo-sourced IL-1Ra and sTNFR could inhibit the recruitment of circulating leukocytes in inflamed and injured glandular tissues [21,24]. When MSC-derived IL-1Ra binds to the IL-1 receptor (IL-1R) on endothelial cells of lacrimal and salivary glands, it blocks the interaction of IL-1β with IL-1R, attenuating the pro-inflammatory signals elicited from activated IL-1R [24]. Likewise, MSC-derived sTNFR prevents TNF-α from binding to its receptor on endothelial cells, acting as an anti-inflammatory agent [21]. Therefore, it is possible that various pro-inflammatory events initiated by IL-1β:IL-1R or TNF:TNFR binding, including the synthesis of immune cell-attracting chemokines followed by enhanced influx of circulating leucocytes in inflamed lacrimal and salivary glands of patients with pSS, might be inhibited by MSC-derived IL-1Ra and sTNFR [21,24].

In addition to IL-1Ra and sTNFR, MSC-derived TGF-β might play a crucial role in mitigating T cell-driven inflammatory responses in patients with pSS [25]. MSC-derived TGF-β inhibits the Janus kinase (Jak)-Stat signaling pathway in IFN-γ-producing Th1 cells and IL-17-producing Th17 cells, causing cell cycle arrest at the G0/G1 phase [21]. Accordingly, it is possible that TGF-β from MSCs might reduce the proliferation and expansion of inflammatory Th1 and Th17 lymphocytes, significantly lowering their numbers in the inflamed eyes, lacrimal, and salivary glands of patients with pSS [25].

Similarly to TGF-β, MSC-derived IL-10 is responsible for the MSC-dependent generation of an immunosuppressive environment in the inflamed and injured tissues [21]. IL-10 promotes the development of tolerogenic DCs, which support the differentiation of naïve CD4+ T cells into immunosuppressive FoxP3+Tregs [26]. However, chronic inflammation can weaken Treg-mediated immunosuppression [27]. The continuous activation of T cell receptors leads to the phosphorylation and activation of PKB/Akt and the mammalian target of rapamycin (mTOR) pathways in resting Tregs [27]. This activation alters Tregs’ immunoregulatory functions, pushing them toward a pro-inflammatory Th17-like phenotype, resulting in the increased production of inflammatory cytokines IL-17 and IL-22 [27]. MSC-derived IDO lowers tryptophan levels in the inflamed environment, activating the general control nonderepressible 2 (GCN2) kinase [21,27]. This activation inhibits the Akt/mTOR signaling pathway in Tregs, preventing their transformation into inflammatory Th17 cells [27]. In this way, MSC-derived IDO may be used for the suppression of Th17-driven inflammation in the inflamed eyes and lacrimal and salivary glands of patients with pSS [28].

**Figure 1:**
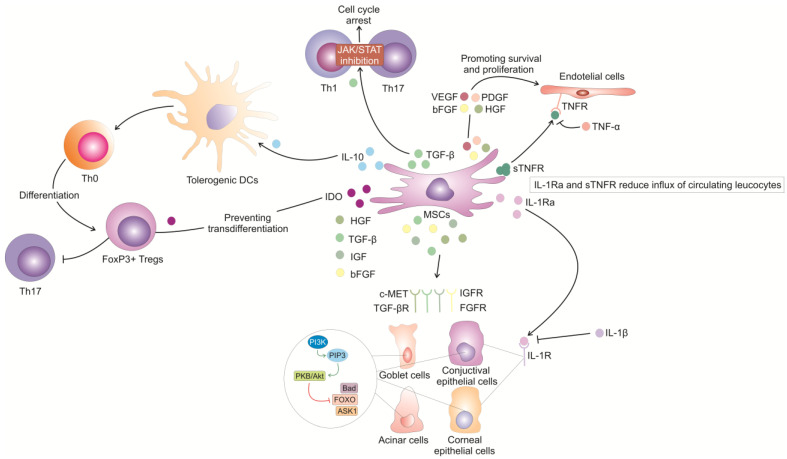
The effects of MSC-derived immunomodulatory and growth factors on survival, migration, phenotype and function of immune cells and parenchymal cells in inflamed and injured lacrimal and salivary glands. MSC-derived HGF, TGF-β, IGF and bGFGF bind to their receptors on injured cells of the ocular surface, salivary and lacrimal glands, activate PI3K kinase which produces PIP3 that induces activation of Akt kinase. Akt then phosphorylates and inhibits pro-apoptotic factors Bad, FOXO, and ASK1, thereby preventing apoptosis of injured cells in the corneas, salivary and lacrimal glands. When MSC-derived IL-1Ra binds to IL-1R on endothelial cells of lacrimal and salivary glands, it blocks the interaction of IL-1β with IL-1R. Similarly, MSC-sourced sTNFR prevents TNF-α from binding to its receptor on endothelial cells. In this way, MSC-derived IL-1Ra and sTNFR reduce influx of circulating leucocytes in inflamed lacrimal and salivary glands of pSS patients. MSC-derived TGF-β prevents proliferation and expansion of inflammatory, IFN-γ-producing Th1 and IL-17-producing Th17 cells by inhibiting Jak-Stat signaling pathway, causing cell cycle arrest in these inflammatory cells. MSC-derived IL-10 promotes the development of tolerogenic DCs which support differentiation of naïve CD4+ T cells into immunosuppressive FoxP3+ Tregs. MSC-sourced IDO prevents transdifferentiation of Tregs in Th17 cells and increases Tregs/Th17 cell ratio in inflamed and injured lacrimal and salivary glands, crucially contributing to the creation of immunosuppressive environment. MSC-sourced pro-angiogenic factors (VEGF, angiopoietin, HGF, bFGF, PDGF) promote survival and proliferation of endothelial cells, increase blood flow and provide nutrients for enhanced regeneration of injured cells in the corneas, salivary and lacrimal glands.

In addition to immunosuppressive molecules, MSCs produce a large number of growth factors that could prevent apoptosis and improve the viability of injured cells of the ocular surface, salivary, and lacrimal glands, enhancing their survival, proliferation, and differentiation [Figure 1] [29]. When MSC-derived HGF, TGF-β, insulin-like growth factor (IGF), and basic fibroblast growth factor (bGFGF)) bind to their receptors on injured cells, they activate phosphatidylinositol 3-kinase (PI3K), which produces phosphatidylinositol (3,4,5)-trisphosphate (PIP3), subsequently activating PKB/Akt [29]. Akt then phosphorylates and inhibits various pro-apoptotic factors such as Bad, FOXO, and ASK1, thereby preventing apoptosis of injured cells in the corneas and salivary and lacrimal glands of patients with pSS [29,30].

In addition to growth factors, MSCs could also provide vascular support to damaged cells through the activity of MSC-sourced pro-angiogenic factors (VEGF, angiopoietins, HGF, bFGF, and PDGF), which promote angiogenesis, enhance blood flow to the injured areas, and provide nutrients for cell regeneration [31].

The therapeutic potential of MSCs in pSS treatment may also rely on their enormous potential for differentiation [8]. When MSCs are cultured under specific conditions, they may differentiate into cells of endodermal and ectodermal origins, including corneal epithelial cells (CECs), conjunctival epithelial cells (ConECs), goblet cells (GCs), and acinar cells (ACs) [32]. The differentiation of MSCs into CECs and ConECs is governed by a variety of signaling pathways, transcription factors, miRNAs, and regulatory molecules. Epidermal growth factor (EGF) and keratinocyte growth factor (KGF) play pivotal roles in promoting the proliferation and differentiation of epithelial cells [32]. These factors can enhance the signaling pathways that lead to corneal epithelial specification from MSCs. The activation of TGF-β/Smad and Delta/Notch signaling can guide the MSCs toward an epithelial lineage by up-regulating the expression of transcriptional factors p63 and Krüppel-like factor 4 (KLF4), which control the expression of genes that are associated with corneal epithelial lineage commitment [32]. Similarly, specific miRNAs, such as miR-145 and miR-205, may target these transcription factors, promoting differentiation of MSCs in CECs and ConECs [32].

The differentiation of MSCs into ACs within the lacrimal glands involves activation of the Wnt/β-catenin pathway, which stabilizes β-catenin, allowing it to enter the nucleus and initiate the transcription of genes essential for acinar cell development, such as aquaporin 5 (Aqp5) and mucin 5AC (MUC5AC) [32] Concurrently, FGF (fibroblast growth factor) signaling promotes MSC proliferation and differentiation through the activation of FGFRs, stimulating downstream pathways that enhance acinar-specific gene expression [32]. The interplay of these pathways, including the involvement of transcription factors HNF6 and GATA3, orchestrates the precise differentiation of MSCs into functional acinar cells, crucial for maintaining tear film stability and ocular surface health [32].

The differentiation of MSCs into GCs involves the activation of the Notch signaling pathway, which promotes the expression of goblet cell-specific marker MUC5AC [32]. Furthermore, by activating the signal transducer and activator of the transcription (STAT-6) pathway, IL-4 and IL-13 could promote the activation of transcription factors GATA3 and HNF1β, which enhance expression of goblet cell-specific genes in MSCs, facilitating differentiation of MSCs in goblet cells [32]. In this way, MSCs could contribute to the restoration of the epithelial barrier in the injured corneas of patients with DED [8,32]. Accordingly, the transplantation of MSC-derived CECs, ConECs, GCs, and ACs may be used for the regeneration of injured tissues in patients suffering from severe pSS [32].

By using a rat model of Sjogren’s syndrome-associated DED, Imaizumi and colleagues demonstrated that the ocular instillation of human AT-MSC-derived conditioned medium (AT-MSC-CM) suppressed immune cell-driven damage of CECs and significantly improved corneal barrier function [33]. AT-MSC-CM, in TGF-β- and JAK/STAT-dependent manners, attenuated the down-regulated expression of pro-inflammatory genes (TNF-α, IL-1, and IL-6) and increased the expression of barrier function-related proteins (TJP1, CDH1, and MUC16) in CEC [33]. In this way, AT-MSC-CM might prevent CEC-driven inflammation, improve thinning, and restore integrity and barrier function of injured corneal epithelium [33].

In addition to their capacity to enhance corneal re-epithelization, MSCs have the potential to promote the repair and regeneration of vital organs, kidneys, and lungs, which are, due to systemic inflammation, frequently injured in patients suffering from severe pSS [7,8]. Results obtained in animal models showed that transplanted MSCs could successfully integrate into the renal architecture and may differentiate into podocytes and tubular epithelial cells, promoting the repair of damaged nephrons and enhancing the filtration capabilities of injured kidneys [32]. MSC-derived VEGF may enhance angiogenesis and improve blood supply to ischemic areas of damaged kidney, while MSC-sourced HGF could stimulate the proliferation of renal cells, protecting them from apoptosis [29,31]. By suppressing the proliferation of fibroblasts and by promoting matrix remodeling, MSCs may prevent renal fibrosis, which is frequently observed in patients with severe pSS as a complication of chronic kidney inflammation [31].

Similarly to their effects on the kidneys, MSCs in the lungs can contribute to the regeneration of damaged alveolar cells [7,31]. They can differentiate into type II alveolar epithelial cells, which play a crucial role in maintaining the alveolar–capillary barrier and ensuring proper gas exchange [8,9]. MSCs may improve lung function by enhancing the production of surfactant proteins, which are vital for reducing surface tension in the alveoli [8,9]. In this way, MSCs could prevent atelectasis in the lungs of patients with pSS [8,9]. Importantly, MSC-derived anti-inflammatory and immunosuppressive cytokines (IL-1Ra, sTNF, and IL-10) may inhibit the activation of immune cells and may reduce their recruitment in the lungs of patients with pSS, preventing the aggravation of lung injury and inflammation [23,29]. Additionally, MSCs can counteract the fibrotic changes in inflamed lung tissue [7,8]. By inhibiting the activation of myofibroblasts and by promoting the secretion of matrix metalloproteinases, MSCs can help dissolve excess fibrous tissue, thereby improving lung compliance and functionality [7,31].

MSCs are found in various tissues, including bone marrow (BM), adipose tissue (AT), dental pulp (DP), dental follicle (DF), umbilical cord (UC), amniotic fluid (AF), and from these tissues may be easily isolated and transplanted in patients with pSS [34]. Importantly, MSCs can be transplanted into HLA-mismatched recipients with a minimal risk of rejection. MSCs are considered “immunoprivileged” cells due to their remarkable ability to avoid detection and attack by the immune system during allogeneic transplantation [35]. They have low levels of HLA class I molecules and completely lack HLA class II molecules, both of which are essential for the activation of an allogeneic response [35]. Moreover, MSCs produce co-inhibitory molecules such as programmed death-ligand 1 (PD-L1) and B7-H4 and promote the generation and expansion of immunosuppressive Tregs and MDSCs, which is vital for maintaining immune tolerance in transplanted tissues [35]. Accordingly, a large number of experimental studies demonstrated that both autologous and allogeneic MSCs may be used in cell-based targeted therapy of pSS [9,10].

## 4. MSC-Based Modulation of T and B Cell-Driven Inflammation in Salivary and Lacrimal Glands of Experimental Animals

**Figure 2:**
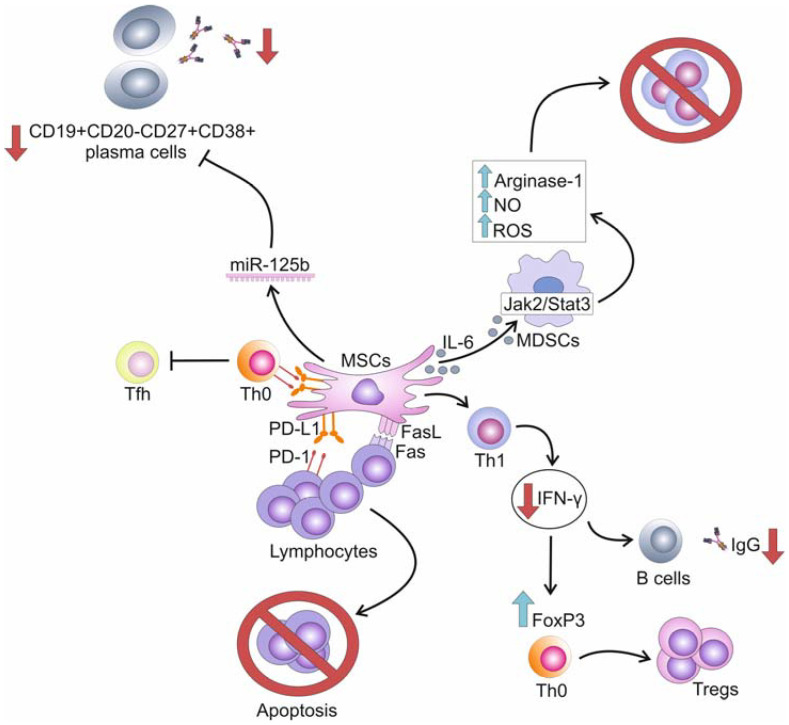
Molecular mechanisms responsible for the suppression of T:B cell cross-talk in inflamed glandular tissues. MSCs induce programmed cell death of rapidly proliferating lymphocytes in PD-L1 and Fas-dependent manner, reduce production of IFN-γ in activated Th1 cells, prevent IFN-γ-dependent synthesis of auto-reactive IgG in B cells and favored expression of FoxP3 in naïve T cells, enabling generation and expansion of immunosuppressive Tregs. MSCs, in PD-L1-dependent manner, prevent differentiation of naïve CD4+T cells in follicular T cells. By delivering IL-6, MSCs activate the Jak2/Stat3 pathway in MDSCs which led to the enhanced production of Arginase-1, NO and ROS. These mediators induce oxidative stress of inflammatory T cells and reduce their number in lachrymal and salivary glands. MSC-sourced miR-125b inhibits expansion of CD19+CD20-CD27+CD38+ plasma cells and reduces production of autoantibodies, attenuating antibody-dependent pathological changes in the inflamed salivary and lacrimal glands.

Several research groups demonstrated that MSCs and their exosomes attenuated pSS-related pathological changes in salivary and lacrimal glands and prevented the progression of the disease by suppressing T and B cell-driven inflammatory responses in experimental animals [Figure 2] [36,37,38,39,40,41,42].

By using a murine model of pSS, Cong and coworkers showed that the MSC-based suppression of IL-17 producing γδ T cells improved the saliva flow rate, attenuated the pathological injury of salivary glands, and inhibited pulmonary inflammation in experimental animals [36]. Shi and colleagues showed that MSCs significantly attenuated the production of IL-12 and prevented the IL-12-based activation of T-bet transcriptional factor in naïve T cells. In this way, MSCs inhibited the generation of T-bet+ and IFN-γ-producing Th1 cells and suppressed the Th1 cell-dependent activation of autoreactive B cells, thus alleviating the production of IgG autoantibodies in MSC-treated pSS mice [37]. Yao and colleagues showed that MSC-derived interferon beta (IFN-β) induced the synthesis of IL-27 in DCs. By producing IL-27, MSC-primed DCs suppressed the proliferation of Th17 lymphocytes, induced the expansion of Tregs, and restored Tregs/Th17 balance in the inflamed glandular tissues of pSS animals, leading to the attenuation of pSS-related signs and symptoms [38].

Similarly to these findings are results reported by Zou and colleagues, who showed that intravenously injected UC-MSCs and UC-MSC-Exos increased the Tregs/Th17 cells ratio in inflamed salivary and lacrimal glands of experimental animals, alleviating pSS-related signs and symptoms [39]. Importantly, the phenotype and function of T cells were significantly altered after exposure to UC-MSCs or UC-MSC-Exos. Down-regulated mRNA expression and the reduced production of inflammatory cytokines (IFN-γ and IL-6) and increased synthesis of immunosuppressive IL-10, PGE2, and TGF-β were observed in MSC or MSC-Exos-exposed T cells [39]. An increase in the Tregs/Th17 cell ratio completely altered the cytokine milieu (attenuated concentrations of inflammatory, pro-Th17 cytokines (TNF-α, IL-6, IL-2, and IL-17) and increased concentrations of anti-inflammatory Treg-derived IL-10 and TGF-β), which improved pSS-related symptoms, prevented the development of extraglandular manifestations of the disease, changed the composition of gut microbiota, restored gut homeostasis, and enhanced the recovery of UC-MSC and UC-MSC-Exo-treated mice [39].

Li and colleagues showed that Exos, which were obtained from labial gland-derived MSCs (LG-MSC-Exos), favored the differentiation of patients with pSS’s naïve T cells in TGF-β-producing Tregs and prevented their trans-differentiation in inflammatory and IL-17-producing Th17 cells [40]. Additionally, LG-MSC-Exos reduced serum levels of Th17-related inflammatory cytokines (IL-6 and IL-17), elevated the levels of the immunosuppressive TGF-β, reduced the presence of Th17 cells, induced the expansion of Tregs and improved tear secretion in a murine model of pSS, alleviating dry eye-related symptoms [40]. Similar findings were reported by Ma and colleagues, who analyzed the effects of UC-MSC-Exos on different peripheral blood lymphocyte subsets of patients with pSS and observed that UC-MSC-Exos inhibited proliferation of CD4+ T cells, attenuated T cell-driven inflammation, and restored the Tregs/Th17 balance [41]. The main mechanism of UC-MSC-Exos-based effects was the inhibition of autophagy in activated T cells, which was evidenced by the down-regulated expression of BECLIN1 and LC3II in UC-MSC-Exo-exposed CD4+T cells [41]. This is in line with the results recently obtained by Shin and colleagues, who used the pSS murine model to demonstrate that the beneficial effects of MSCs in the attenuation of ocular inflammation and dry eye-related symptoms were due to the MSC-based inhibition of autophagy in lacrimal glands [42]. The subconjunctival injection of MSCs reduced the expression of autophagy-related genes (ATG5, LC3B-II) and inhibited mTOR-driven signaling in the eyes of experimental animals [42]. Additionally, MSCs suppressed the production of inflammatory cytokines and prevented the expansion of activated B cells, which improved tear secretion and attenuated corneal injury in MSC-treated pSS mice [42].

The T-cell immunoglobulin (Ig) mucin family containing molecule 3 (Tim-3), which regulates the production of anti-inflammatory cytokines, is down-regulated in T cells of patients with pSS [43]. Additionally, it induces epithelial–mesenchymal transition (EMT) and causes the dysfunction of exocrine gland epithelial cells in patients with pSS [43]. Sun and colleagues showed that the MSC-based modulation of Tim-3 in patients with pSS’s T cells was mainly responsible for the beneficial effects of MSCs in the attenuation of pSS [44]. The MSC-dependent up-regulation of Tim-3 on T cells was followed by the increased production of immunosuppressive cytokines [44]. Additionally, MSCs attenuated submandibular gland dysfunction by reversing EMT through down-regulated Tim-3 expression. Transplanted MSC ameliorated pathological changes in the submandibular glands, attenuated T cells driven-inflammation and fibrosis, and alleviated all pSS-related signs and symptoms [44].

DF-MSCs, DP-MSCs, and MSCs, which were isolated from human exfoliated deciduous teeth (SHED) or olfactory lamina propria (OLP-MSCs), suppressed the proliferation of activated CD4^+^ T lymphocytes and prevented the differentiation of naïve B cells in antibody-secreting plasma cells [45,46,47]. The immunomodulatory effects of MSCs were based on their capacity to regulate the viability, proliferation, and activation of T cells and to modulate their cross-talk with B cells [45,46,47]. MSCs induced programmed cell death of rapidly proliferating lymphocytes in PD-L1 and Fas-dependent manner, reduced production of IFN-γ in activated Th1 cells, prevented IFN-γ-dependent synthesis of auto-reactive IgG in B cells, and favored expression of FoxP3 in naïve T cells, enabling generation and expansion of immunosuppressive Tregs in secondary lymph organs [45,46,47]. Similarly, OLP-MSC-Exos in a PD-L1-dependent manner prevented the differentiation of naïve CD4+T cells in FTH and inhibited the FTH-dependent generation of autoreactive B cells in lymphoid folicules [48]. In this way, OLP-MSC-Exos reduced the production of autoantibodies and ameliorated disease progression in pSS mice [48]. Additionally, OLP-MSCs-Exos enhanced the synthesis of reactive oxygen species (ROS) in MDSCs and improved their immunosuppressive ability [48]. By delivering IL-6, OLP-MSC-Exos activated the Jak2/Stat3 pathway in MDSCs, which led to the enhanced production of Arginase-1, nitric oxide (NO), and ROS. These mediators induced oxidative stress of inflammatory T cells and reduced their number in lachrymal and salivary glands of OLP-MSC-Exo-treated pSS mice, attenuating disease progression [48]. Similar findings were obtained by Tian and colleagues, who showed that BM-MSCs, in a TGF-β-dependent manner, induced increased synthesis of Arginase-1 and NO in MDSCs, enhancing their immunosuppressive properties [49]. MDSCs from BM-MSC-treated pSS mice efficiently attenuated the expression of co-stimulatory molecules on B cells and impaired their cross-talk with auto-reactive T cells, alleviating pSS-related signs in experimental animals [49]. In line with these findings are results obtained by Xing and colleagues, who showed that intravenously injected LG-MSC-Exos efficiently attenuated the progression of pSS in experimental mice by modulating the activation of B cells in miR-125b-dependent manner [50]. PR domain zinc finger protein 1 (PRDM1) regulates the differentiation of B cells into plasma cells and controls the secretion of antibodies, playing an important role in the development and progression of pSS [51]. By affecting the expression of the PR domain zinc finger protein 1 (PRDM1) gene, which regulates the differentiation of B cells and controls the secretion of antibodies, LG-MSC-Exo-sourced miR-125b inhibited the expansion of CD19+CD20-CD27+CD38+ plasma cells and reduced the production of autoantibodies [50]. The LG-MSC-Exos-dependent suppression of autoreactive plasma cells prevented the generation of the systemic inflammatory response, reduced the influx of circulating leukocytes in inflamed salivary glands, and restored saliva secretion in LG-MSC-Exo-treated pSS mice [50].

## 5. Tissue Source and the Route of Injection Affect Therapeutic Effects of MSCs

Although MSCs, which are isolated from various tissues, share numerous morphological and functional characteristics, the microenvironment in which MSCs are grown modulates some of their immunomodulatory properties [52,53]. Ogata and co-workers observed that the conditioned medium (CM), which was obtained from DP-MSCs, contained higher concentrations of immunosuppressive cytokines (TGF-β and IL-10) than BM-MSC-CM [52]. Accordingly, DP-MSC-CM more efficiently suppressed T cell-driven inflammation in salivary glands, increased salivary flow rate, and attenuated pSS-related symptoms in experimental animals than BM-MSC-CM [52]. Similar findings were reported by Matsumura-Kawashima and colleagues, who demonstrated that DP-MSC-CM more efficiently than BM-MSC-CM suppressed the production of IL-17 and IL-4, increased the synthesis of IL-10 and TGF-β, and induced an increase in the Tregs/Th17 cell ratio in inflamed tissues of pSS mice [53].

Huang and co-workers revealed that the migratory characteristics of BM-MSCs were impaired in patients with pSS compared to healthy volunteers [54]. They observed that the expression of the motility-related gene CFL1 and chemokine receptor CCR1 were significantly decreased in pSS-derived BM-MSCs. Down-regulated CFL1 and CCR1 expression attenuated the capacity of MSCs to migrate in inflamed and injured salivary and lacrimal glands, reducing their therapeutic potential [54]. Huang and colleagues engineered CFL1-overexpressing BM-MSCs. Lentivirus-mediated CFL1 overexpression promoted the migration of pSS patient-derived BM-MSCs in the injured tissues of experimental animals and restored their capacity for the suppression of immune cell-driven inflammation [54].

The route of injection significantly affects the therapeutic efficacy of transplanted MSCs [55]. MSCs that were locally transplanted into injured and inflamed glandular tissues mainly differentiated in injured epithelial cells, supporting enhanced tissue regeneration, while the beneficial effects of systemically infused MSCs mainly relied on their capacity to suppress systemic inflammatory response in paracrine and endocrine manners [55]. Du and colleagues showed that intravenously infused MSCs migrated to the spleen and the liver and not to the inflamed submandibular glands of pSS mice. MSCs directed naive T cells toward Tregs and suppressed the generation of Th1 and Tfh cells in the spleens [56]. Although MSCs did not infiltrate the inflamed glandular tissues, they directed naive T cells toward Tregs and suppressed the generation of Th1 and Tfh cells in the spleens [56]. In this way, MSCs indirectly, through the suppression of T cell-driven inflammatory response, prevented ATG5 and Beclin-1-dependent autophagy and the apoptosis of epithelial cells in inflamed submandibular glands, importantly contributing to the enhanced repair of injured glandular issues [56]. Similar findings were reported by Genç et al., who showed that intraperitoneally injected DF-MSCs suppressed the production of inflammatory cytokines (IFN-γ and IL-17) in T cells, attenuated the generation and expansion of CD4+Th1 and Th17 lymphocytes in the spleen, and ameliorated systemic inflammatory response in pSS mice but did not engraft in injured salivary and lacrimal glands [55]. On the contrary, the beneficial effects of DF-MSCs, which were transplanted directly into injured submandibular or lacrimal glands of pSS mice, mainly relied on their regenerative properties. DF-MSCs successfully engrafted in inflamed glands and differentiated in glandular epithelial cells, ameliorating glandular dysfunction [55].

## 6. Therapeutic Potential of MSCs in the Treatment of pSS: Evidence from Pilot Clinical Studies

A double-blinded randomized clinical trial (NCT04615455) that investigated the therapeutic potential of allogeneic AT-MSCs in the treatment of DED in patients with pSS has been recently completed in Denmark [57]. A single transconjunctival injection of allogeneic AT-MSCs, which were isolated from healthy individuals, significantly improved subjective and objective signs and symptoms of dry eye disease in patients with pSS, as evidenced by the reduced Ocular Surface Disease Index (OSDI) score, increased tear break-up time, improved ocular surface staining, tear osmolarity, and Schirmer test score, which were observed in AT-MSC-treated patients with pSS compared to those patients who received lubricating eye drops, indicating the efficacy of locally injected MSCs for the treatment of dry eye-related symptoms in patients with pSS [57].

Similar findings were reported in a study that investigated the therapeutic potential of derived Multiple Allogeneic Proteins Paracrine Signaling (d-MAPPS^TM^), an ophthalmic solution that contains AF-MSC-derived secretome. Eye discomfort and pain were successfully reduced in 131 patients who received the d-MAPPS^TM^ ophthalmic solution [58]. d-MAPPS^TM^ managed to alleviate eye inflammation, support tear stability, and prevent ocular surface epithelial damage, crucially contributing to the enhanced repair and regeneration of the ocular surface epithelial barrier in these patients [58]. d-MAPPS^TM^-treated patients experienced a substantial improvement in all dry eye-related symptoms, as evidenced by the significant drop in VAS and SPEED scores 3 months after starting the treatment, with the maximum improvement seen after a year of treatment, thereby showing its long-term positive impact on reducing eye symptoms in patients with dry eye. Adverse side effects were not reported, indicating that the topical administration of d-MAPPS^TM^ was a safe and efficient therapeutic approach for the treatment of dry eye [58].

In line with these findings are results obtained by Liang and colleagues, who evaluated the safety of MSC infusions in 404 MSC-treated patients with autoimmune diseases, including 72 patients with pSS [59]. During the 43.4 ± 25.9-long follow-up period, the incidence rate of serious infections was 12.9%, where five patients (1.2%) experienced malignancies, and transplantation-related mortality was 0.2%. Liang and colleagues concluded that the incidences of adverse events, whether infections or malignancies, were acceptable in these patients and that the infusion of MSCs should be considered a safe therapy for patients with autoimmune diseases [59].

Based on these promising results, a clinical trial (NCT00953485), which will be conducted in China, plans to investigate the therapeutic potential of allogeneic MSCs in the treatment of patients with pSS who have been resistant to multiple standard treatments. According to the study protocol, MSCs will be intravenously injected (10^6^ cells/kg) in these patients, and their efficacy will be monitored by analyzing the disease activity index, pSS serology, and salivary gland function.

Another clinical trial (NCT04213248), which plans to investigate the therapeutic potential of UC-MSC-Exos for the alleviation of dry eye-related symptoms in patients suffering from ocular graft versus disease (oGVHD), is currently recruiting patients. According to the study protocol, patients with oGVHD will receive artificial tears for 14 days to normalize the baseline, and afterward, UC-MSC-Exo eye drops (10 μg/drop; four times a day) will be administered for two weeks. Changes in the ocular surface disease index, conjunctiva redness scores, tear secretion, tear break time, ocular surface staining, the best corrected visual acuity, and tear meniscus height will be determined during the follow-up of 12 weeks. The first results of this trial are expected in the next two years.

## 7. Conclusions

Results that were obtained in a large number of experimental and in a few clinical studies demonstrated that MSCs should be considered as potentially new therapeutic agents for the treatment of patients with pSS [9,10,36,37,38,39,40,41,42,44,45,46,47,48,49,50,57,58,59]. The therapeutic potential of MSCs mainly relies on their immunosuppressive and regenerative properties [9,10]. MSCs, in juxtacrine and paracrine manners, suppressed the DC-dependent generation of inflammatory Th1 and Th17 lymphocytes, induced the expansion and promoted the immunosuppressive properties of Tregs and MDSCs, impaired the cross-talk between autoreactive T and B cells, and prevented the synthesis and secretion of autoantibodies. Additionally, MSC-derived growth and trophic factors promoted survival and prevented apoptosis of injured cells in inflamed lacrimal and salivary glands, enhancing their repair and regeneration [9,10,36,37,38,39,40,41,42,44,45,46,47,48,49,50,57,58,59].

Despite these encouraging results, it should be noted that all of these findings were obtained either in animal studies or in pilot clinical trials with a small number of recruited patients. Therefore, upcoming large-scale double-blinded randomized clinical trials need to validate these findings before MSCs can be widely offered as new therapeutic agents for the treatment of severe pSS.

## Data Availability

The data that are discussed in this article are presented in cited studies.
